# Hemodynamic Determinants of Elevated Blood Pressure and Hypertension in the Middle to Older-Age UK Population: A UK Biobank Imaging Study

**DOI:** 10.1161/HYPERTENSIONAHA.122.20969

**Published:** 2023-09-07

**Authors:** Ye Li, Emily Chan, Esther Puyol-Antón, Bram Ruijsink, Marina Cecelja, Andrew P. King, Reza Razavi, Phil Chowienczyk

**Affiliations:** 1 Department of Clinical Pharmacology, King’s College London British Heart Foundation Centre, St. Thomas’ Hospital, United Kingdom (Y.L., M.C., P.C.).; 2 School of Bioengineering and Imaging Science, King’s College London, United Kingdom (E.C., E.P.-A., B.R., A.P.K., R.R.).

**Keywords:** aortic distensibility, blood pressure, cardiac output, hemodynamic, hypertension, systemic vascular resistance

## Abstract

**BACKGROUND:**

Increased systemic vascular resistance and, in older people, reduced aortic distensibility, are thought to be the hemodynamic determinants of primary hypertension but cardiac output could also be important. We examined the hemodynamics of elevated blood pressure and hypertension in the middle to older-aged UK population participating in the UK Biobank imaging studies.

**METHODS:**

Cardiac output, systemic vascular resistance, and aortic distensibility were measured from cardiac magnetic resonance imaging in 31 112 (distensibility in 21 178) participants (46.3% male, mean age±SD 63±7 years). Body composition including visceral adipose tissue volume and abdominal subcutaneous adipose tissue volume were measured in 19 645 participants.

**RESULTS:**

Participants with higher blood pressure had higher cardiac output (higher by 17.9±26.6% in hypertensive compared with those with optimal blood pressure) and higher systemic vascular resistance (higher by 11.4±27.9% in hypertensive compared with those with optimal blood pressure). These differences were little changed after adjustment for body size and adiposity. The contribution of cardiac output relative to systemic vascular resistance was more marked in younger compared with older subjects. Aortic distensibility decreased with age and was lower in participants with higher compared with lower blood pressure but with a greater difference in younger compared with older subjects.

**CONCLUSIONS:**

In the middle to older-aged UK population, cardiac output plays an important role in contributing to elevated mean arterial blood pressure, particularly in younger compared with older subjects. Reduced aortic distensibility contributes to a rise in pulse pressure and systolic blood pressure at all ages.

**NOVELTY AND RELEVANCE**

**What Is New?**

In the middle to older-aged UK population, cardiac output plays an important role in contributing to elevated mean arterial blood pressure, particularly in younger compared with older subjects. Reduced aortic distensibility contributes to a rise in pulse pressure and systolic blood pressure at all ages.

**What Is Relevant?**

Understanding the hemodynamic determinants of hypertension may identify new mechanisms and targets to prevent and treat hypertension.

**Clinical/Pathophysiological Implications?**

The pathogenesis of hypertension may be more strongly linked to factors that elevate cardiac output than previously recognized; reduced aortic distensibility may be an important cause of hypertension at all ages. Interventions that target these factors may be effective in preventing and treating hypertension.

Hemodynamic determinants of blood pressure (BP) include cardiac output (CO), peripheral systemic vascular resistance (SVR), and the distensibility of the aorta (AoD).^1,2^ CO and SVR determine mean arterial BP (MAP) as the product of CO and SVR, whereas AoD (inversely related to aortic stiffness) is a major determinant of pulse pressure (PP).^3,4^ Other properties influencing PP are ventricular dynamics and peak aortic flow velocity,^5^ and when measured in the periphery of the circulation, amplification of peripheral PP over central (aortic root) PP due to the phenomenon of pressure wave amplification.^5,6^ The conventional view of the hemodynamics of hypertension is that hypertension results from an elevated SVR^7^ and, particularly in older subjects, reduced AoD.^2^ However, this conclusion derives mainly from studies comparing groups of hypertensive compared with normotensive individuals. In population studies, particularly in younger subjects, raised CO contributes to elevated BP.^8–10^

The objective of the present study was to examine the hemodynamic determinants of raised BP and hypertension in the middle to older-age UK population participating in the UK Biobank imaging study. This provides detailed characterization of cardiovascular properties via cardiac magnetic resonance imaging (MRI), the most accurate modality for measurement of CO. It also provides measures of body composition including visceral fat and abdominal subcutaneous fat mass allowing the potential influence of body composition on CO to be explored.

## METHODS

### Data Availability

The raw data and images used in this study are available from UK Biobank and hemodynamic variables obtained from the images will be available from UK Biobank (https://www.ukbiobank.ac.uk). UK Biobank is a large prospective cohort study recruiting more than half a million participants aged 40 to 69 years from the general population between 2006 and 2010. The UK Biobank imaging study aims to scan 100 000 of the original participants and started in 2014. In the present study, we included data from 48 000 participants completed before February 2021 which included detailed cardiovascular magnetic resonance (CMR) imaging, excluding those with a diagnosis of cardiac (coronary artery disease or heart failure) or vascular (peripheral artery disease or stroke) disease. All participants provided informed consent at the time of recruitment for their data to be used for research purposes and this research was conducted under UK Biobank Application Number 51560.

### Basic Characterization and BP Measurements

Age, height, weight, smoking status, medical history, and medication were recorded during the imaging visit. Heart rate (HR), brachial systolic BP (SBP), and diastolic BP (DBP) were measured during the same imaging visit using an automated device (Omron 705, OMRON Healthcare Europe B.V. Kruisweg 577 2132 NA Hoofddorp). A manual sphygmomanometer was used if the standard automated device could not be used. An appropriate blood pressure cuff was selected based on the measured circumference of the midpoint of the upper arm. Two consecutive measurements were taken sequentially on the left upper arm by a registered nurse with the participant quietly seated and with no restrictive clothing to the left arm. Mean values from the 2 measurements were used in the analysis. MAP was calculated as DBP+one-third of the PP, with PP the difference between SBP and DBP.

### CO and Aortic Distensibility by CMR

CMR scans were performed using a 1.5 Tesla MAGNETOM Aera scanner (Siemens Healthineers, Erlangen, Germany), as previously described.^11^ UK Biobank’s CMR acquisitions included piloting and sagittal, transverse, and coronal partial coverage of the chest and abdomen. For cardiac function, 3 long-axis cines and a complete short-axis stack of balanced steady-state free precession cines, covering the left ventricle and right ventricle were acquired. Aortic flow was captured by a 2-dimensional phase contrast through-plane acquisition of the ascending aorta, planned using sagittal and coronal left ventricular outflow tract cines. Details of the CMR sequences are summarized elsewhere.^12^ Conventional left ventricular volumetric measures were extracted using a fully automated analysis (AI-CMR^QC^) previously developed and validated in a large subset of the UK Biobank.^13^ Stroke volume (SV) was defined as the difference between left ventricular end-diastolic and end-systolic volume. CO was calculated from SV and HR and SVR from MAP and CO. Aortic flow velocity was extracted using a novel framework, by a fully automated quality-controlled analysis as described elsewhere^14^ and peak aortic flow velocity (Umax) obtained from the aortic flow curve.

AoD was derived from a transverse balanced steady-state free precession cine at the level of the pulmonary trunk and right pulmonary artery as previously described^15^ but with the substitution of an estimate of central aortic PP for brachial PP. An image segmentation network automatically segmented the ascending aorta to determine maximum aortic area (Amax) and minimum aortic area (Amin). A quality control network was used to ensure that change in aortic area over time produced a waveform with physiological morphology (early peak followed by near exponential decay) and to exclude segmentations influenced by motion artifact. The relative change in area, aortic strain (strain) was defined as (Amax−Amin)/Amin. AoD was computed as aortic strain/central aortic PP. Central aortic PP for this calculation was estimated from a waveform derived from a peripheral brachial BP cuff inflated to 70 mm Hg using a Vicorder device and associated software processing (Skidmore Medical, Bristol, United Kingdom).^16^ Brachial cuff–derived waveforms and central aortic PP were obtained immediately before and after the aortic distensibility scan with a mean value from 2 readings used for analysis.^15^

### Body Composition Measurements by Abdominal MRI

Visceral adipose tissue volume (VAT) and abdominal subcutaneous adipose tissue volume (ASAT) were measured as previously described.^17^ A dual-echocardiography Dixon Vibe protocol was used to obtain body composition data, a 6-minute protocol that covered neck to knees and was divided over 6 overlapping slabs of axial 3-dimensional spoiled gradient dual-echocardiography images.^17^ Using the integrated scanner software, fusion of the axial slabs provided a dataset of isolated water and fat volumes. VAT and ASAT were calculated by automatic segmentation using the AMRA Profiler (AMRA Medical AB, Linkoping, Sweden). VAT was defined as the adipose tissue within the abdominal cavity, excluding adipose tissue outside the abdominal skeletal muscles and adipose tissue and lipids within the cavity and posterior of the spine and back muscles. ASAT was defined as the subcutaneous adipose tissue in the abdomen from the top of the femoral head to the top of the thoracic vertebrae T9.

### Statistical Analysis

Participants of each sex were stratified into age and BP categories. Age groups were defined as 45 to 54, 55 to 64, 65 to 74, and 75 to 84 years. BP categories were defined according to seated brachial BP values measured during the imaging assessment visit, previous history of hypertension, and use of antihypertensive therapy. Optimal, normal, and high-normal BPs were defined in the absence of any antihypertensive therapy. Optimal BP was defined as SBP <120 mm Hg and DBP <80 mm Hg, normal BP as SBP 120 to 129 mm Hg and/or DBP 80 to 84 mm Hg, high-normal BP as SBP 130 to 139 mm Hg and/or DBP 85 to 89 mm Hg, hypertensive as SBP≥140 mm Hg and/or DBP≥90 mm Hg, or previously diagnosed hypertension and prescription of antihypertensive therapy.^18^ For a separate analysis, isolated systolic hypertension (ISH) was defined as SBP≥140 mm Hg and DBP<90 mm Hg. Subject characteristics and results are presented as means±SD. Differences in characteristics between different BP groups across all age groups were compared by 1-way ANOVA or (for categorical variables) by χ^2^ test. Two-way ANOVA was used to test for differences in hemodynamic variables between age and BP groups and to test for an interaction between age and BP group. In addition to analysis by BP categories, we examined the relationship of cardiovascular properties (HR, SV, CO, SVR, AoD, aortic strain, and Umax) to BP components (MAP, PP, SBP, and DBP) as a continuum by regression analysis, initially by univariate analysis and then using multivariate analysis to adjust for age, height, and weight. To allow comparison of relative strengths of association all regression coefficients are presented as standardized coefficients. To examine the potential influence of body composition that was not captured by height and weight, we used regression analyses to determine association of cardiovascular properties (HR, SV, CO, SVR, and AoD) with measures of adiposity (VAT and ASAT) after adjusting for age, height and weight (with the analysis performed separately in each sex) and examined the variation of cardiovascular properties according to BP categories after adjustment for VAT and ASAT. Analysis was performed using SPSS version 26 (SPSS, Inc, Chicago, IL), and *P*<0.05 was taken as significant.

## RESULTS

### Characteristics of the Study Population

Characteristics of participants stratified according to BP category are shown in Table 1. There were 31 112 participants in total comprising 4424, 5006, 5644, and 16 038 in the optimal, normal, high-normal, and hypertensive groups respectively. Mean age was 63±7 years, with 46.3% of participants male. In the hypertensive group, 33.0% of the subjects were on antihypertensive therapy. Groups with higher BP were older, had a greater proportion of men, and had higher body mass index.

**Table 1.**
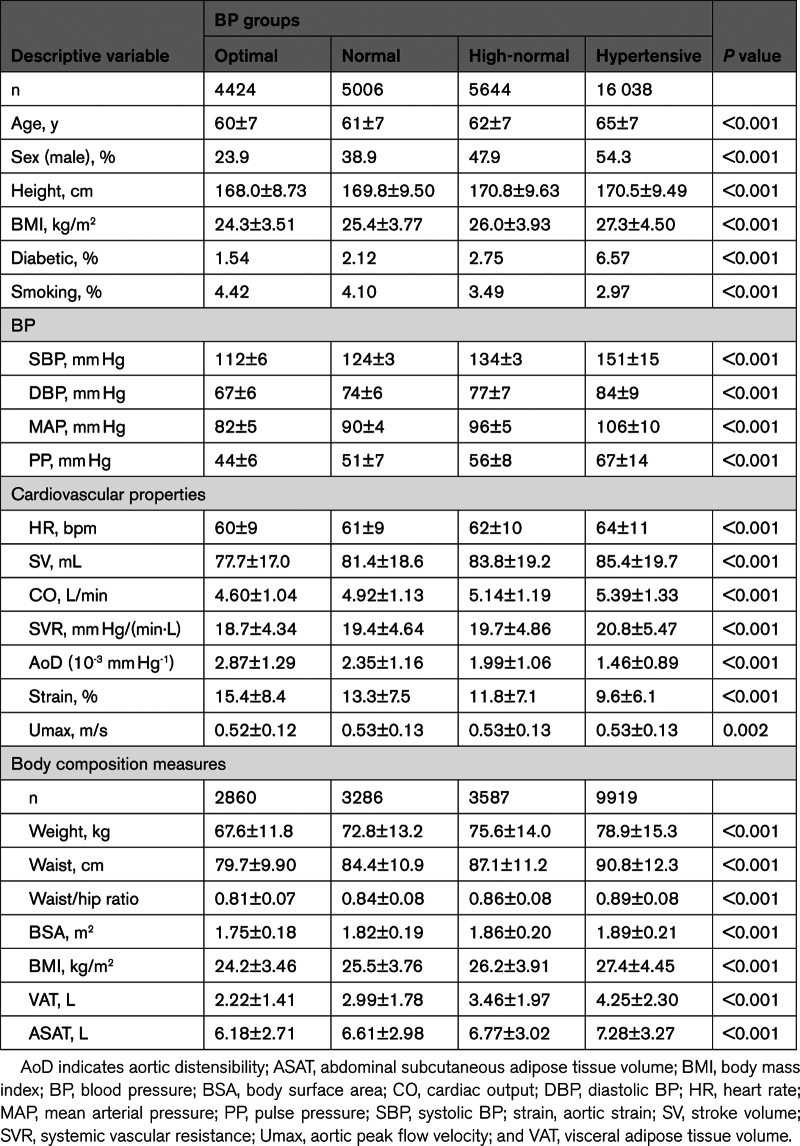
Characteristics of Participants Stratified by BP Group

**Figure 1.**
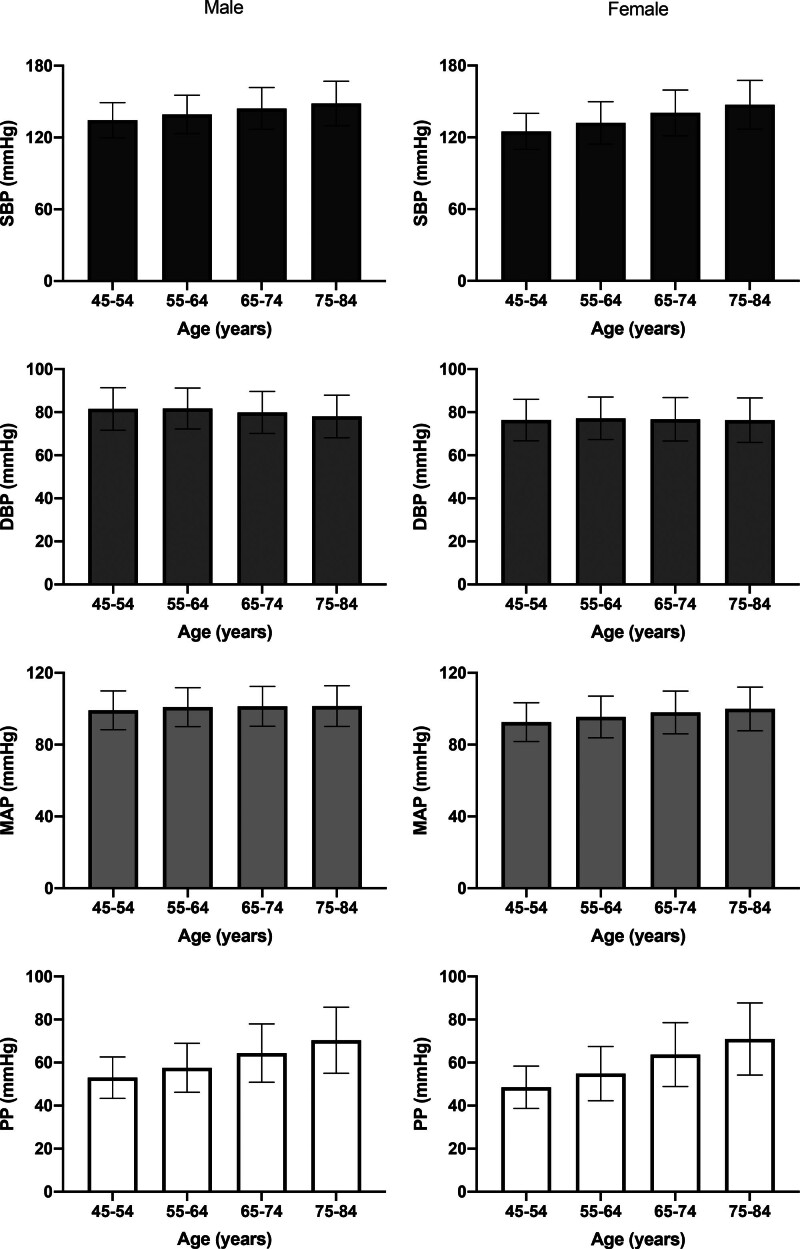
**Mean values of blood pressure components systolic blood pressure (SBP), diastolic blood pressure (DBP), mean arterial pressure (MAP), and pulse pressure (PP) in UK Biobank stratified according to age.** Error bars are SDs.

BP trends with age were as expected in the general population with SBP increasing with age and DBP decreasing with age so that MAP remained approximately constant with age (Figure 1). The overall prevalence of ISH was 31.1% (n=9664). Characteristics of subjects with ISH are shown in Table S1. Compared with normotensive subjects, subjects with ISH were older, were more likely to be male, and had higher BMI.

### Comparison of Cardiovascular Properties Between BP Groups

**Figure 2.**
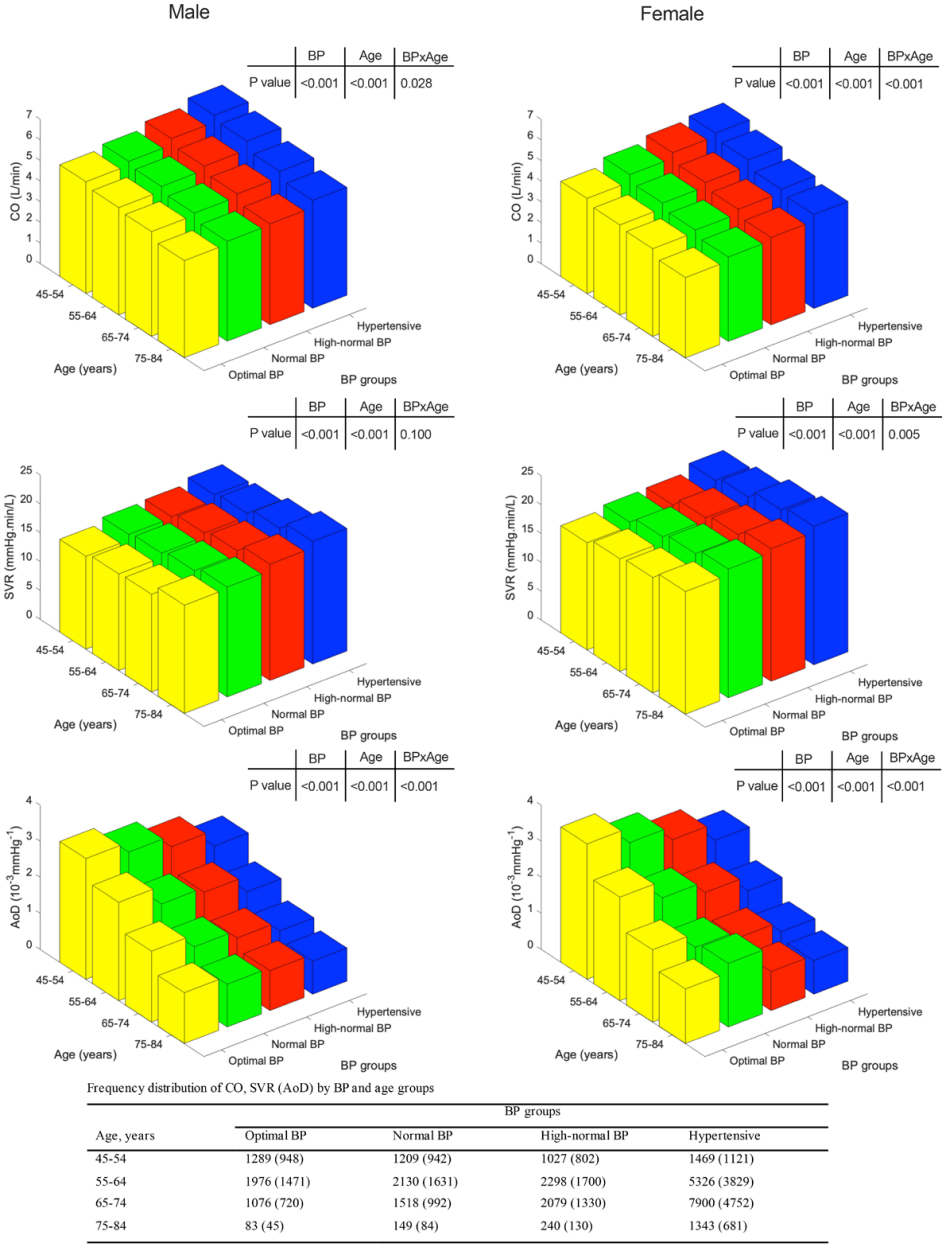
**Hemodynamic variables cardiac output (CO), systemic vascular resistance (SVR), and aortic distensibility (AoD) in UK Biobank stratified according to age and blood pressure (BP) group.** *P* values refer to significant differences in mean values of variables between age and BP groups and the interaction between age and BP (age×BP). Numbers of subjects in each group are presented in the table below the figures, numbers in brackets are for measurements of distensibility. Note that, due to the cross-sectional nature of the data, the number of individuals in higher BP groups increases with age. The plots do not, therefore, provide a measure of the trend of hemodynamic variables with age.

In both sexes, groups with higher compared with lower BP had higher HR and SV (Figure S1). This led to a higher CO in groups with higher compared with lower BP. SVR was also higher in subjects in higher compared with lower BP groups. The variation of cardiovascular properties according to BP group, stratified by age, is shown in in Figure 2. Note that, due to the cross-sectional nature of the data, the number of individuals in higher BP groups increases with age. Figure 2 does not, therefore, provide a measure of the trend of hemodynamic variables with age.

CO was higher by 17.9±26.6% in hypertensive compared with those with optimal BP and SVR was higher by 11.4±27.9% in hypertensive compared with those with optimal BP. There was a significant interaction between BP group and age such that differences in CO between BP groups were more marked at a younger age and differences in SVR in women were more marked at an older age (Figure 2; Figure S2).

Distensibility data were available in 21 178 participants in total comprising 3184, 3649, 3962, and 10 383 in the optimal, normal, high-normal, and hypertensive groups, respectively. Characteristics of these participants were similar to those of the whole study population (Table S2). AoD was slightly lower in men compared with women. In both sexes, AoD decreased with age and was lower in higher compared with lower BP groups with the absolute difference in AoD between BP groups greater in younger compared with older subjects (Figure 2).

### Association of Cardiovascular Properties With BP Components Across the Continuum of the BP Distribution

When examining the association of cardiovascular properties with BP components across the continuum of the BP distribution (Table 2), CO and SVR were associated more strongly with MAP than with PP, and adjustment for age, height, and weight had relatively little effect on the size of the associations. AoD was most strongly associated with PP, and aortic strain and Umax were also significantly associated with PP. Adjustment for age (with or without adjustment for height and weight) had a significant effect on the strength of the associations with PP, decreasing the strength of the associations of AoD and aortic strain with PP (but with the adjusted associations still strong), and increasing the strength of the association of Umax with PP. The association of Umax with PP was less strong than that of AoD with PP.

**Table 2.**
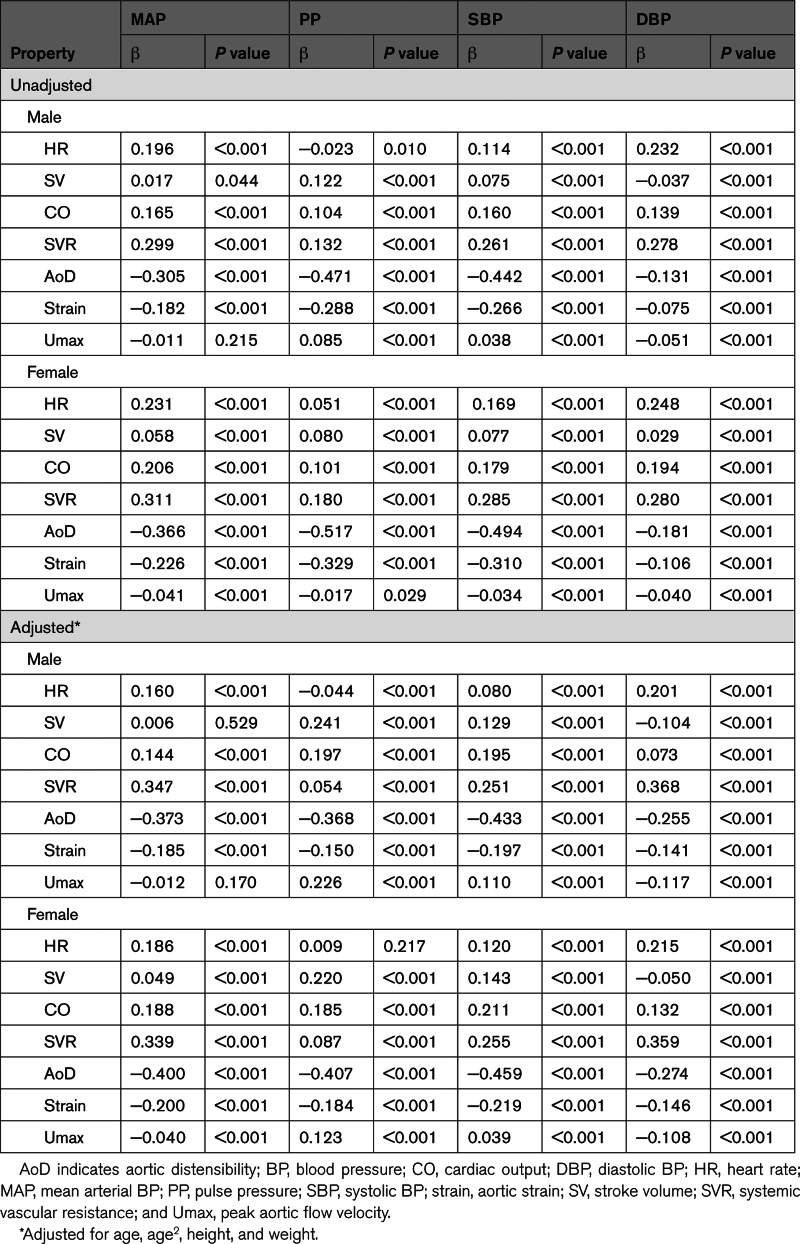
Association of Cardiovascular Properties With BP Components Unadjusted and Adjusted for Age, Height, and Weight

### Comparison of Cardiovascular Properties in Subjects With ISH and Normotensive Subjects

**Figure 3.**
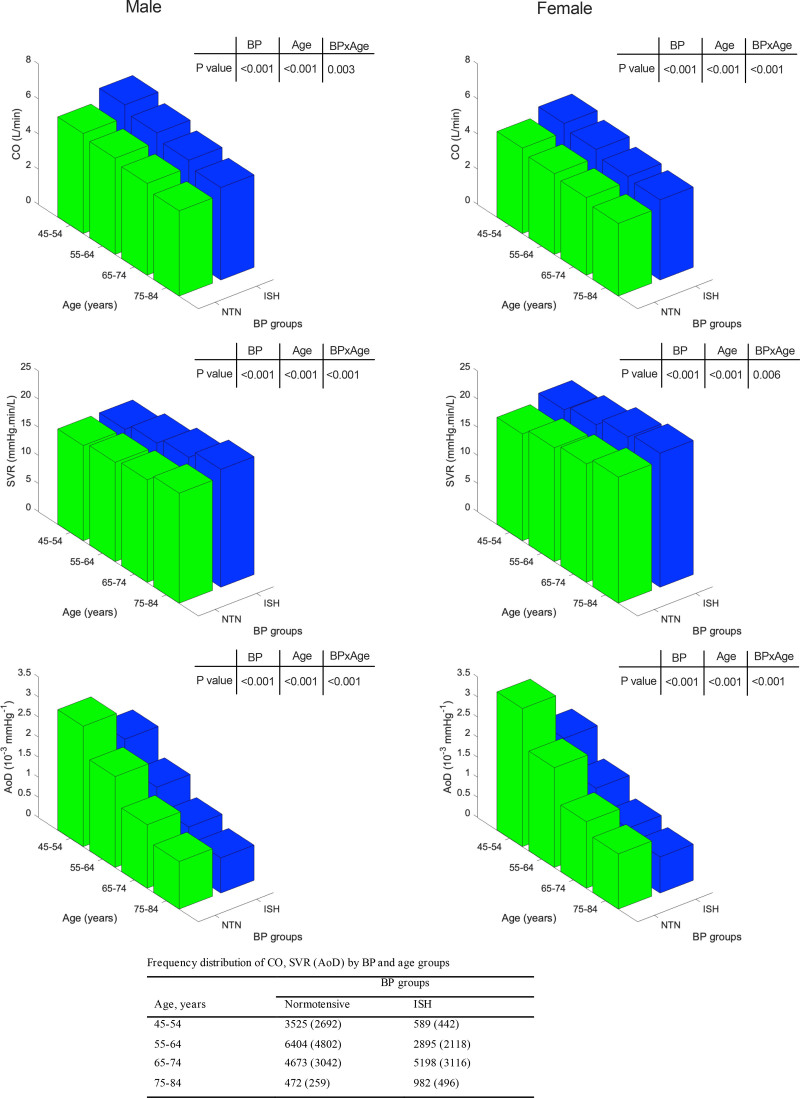
**Hemodynamic variables cardiac output (CO), systemic vascular resistance (SVR), and aortic distensibility (AoD) in UK Biobank stratified according to age and blood pressure (BP) group: normotensive (NTN) and isolated systolic hypertension (ISH).** *P* values refer to significant differences in mean values of variables between age and BP groups and the interaction between age and BP (age×BP). Numbers of subjects in each group are presented in the table below the figures, numbers in brackets are for measurements of distensibility. Note that, due to the cross-sectional nature of the data, the number of individuals in the ISH group increases with age. The plots do not, therefore, provide a measure of the trend of hemodynamic variables with age.

When comparing cardiovascular properties between subjects with ISH and normotensive subjects, CO was increased and AoD decreased with more marked differences in younger compared with older subjects (Figure 3; Table S1; similar considerations with respect to change of cardiovascular properties with age apply to both Figures 2 and 3). The contribution of SVR to ISH varied according to age with different patterns seen in men and women. In men, an increase in SVR was more marked in older compared with younger men, whereas in women the reverse was true. Thus, in younger men with ISH, this was mainly due to raised CO and lower AoD, whereas in younger women it was due to raised SVR and lower AoD. In older subjects (at least up to 74 years) of both sexes, ISH was mainly due to higher SVR and lower AoD.

### Association of Cardiovascular Properties With Body Composition

Body composition measurements were available in 19 645 participants in total comprising 2860, 3284, 3586, and 9915 in the optimal, normal, high-normal, and hypertensive groups, respectively. Characteristics of these subject were similar to those of the whole study population (Table S2). The association of cardiovascular properties with body composition is shown in Table 3. CO was positively associated with height and strongly positively associated with weight and reverse associations were seen with SVR. After adjustment for age, height, and weight, CO was negatively associated with both VAT and ASAT, and SVR was positively associated with VAT and ASAT. The negative association of CO with VAT and ASAT was driven by stronger negative associations of SV with VAT and ASAT than those of HR with VAT and ASAT. AoD was weakly associated with VAT and ASAT. Similar results were seen when height and weight were replaced by body surface area (Table S3). Despite the modest association of cardiovascular properties with measures of adiposity, adjustment for measures of adiposity (and age, height, and weight) had little impact on the relationship between cardiovascular properties and BP categories (Figure 3; Figure S3).

**Table 3.**
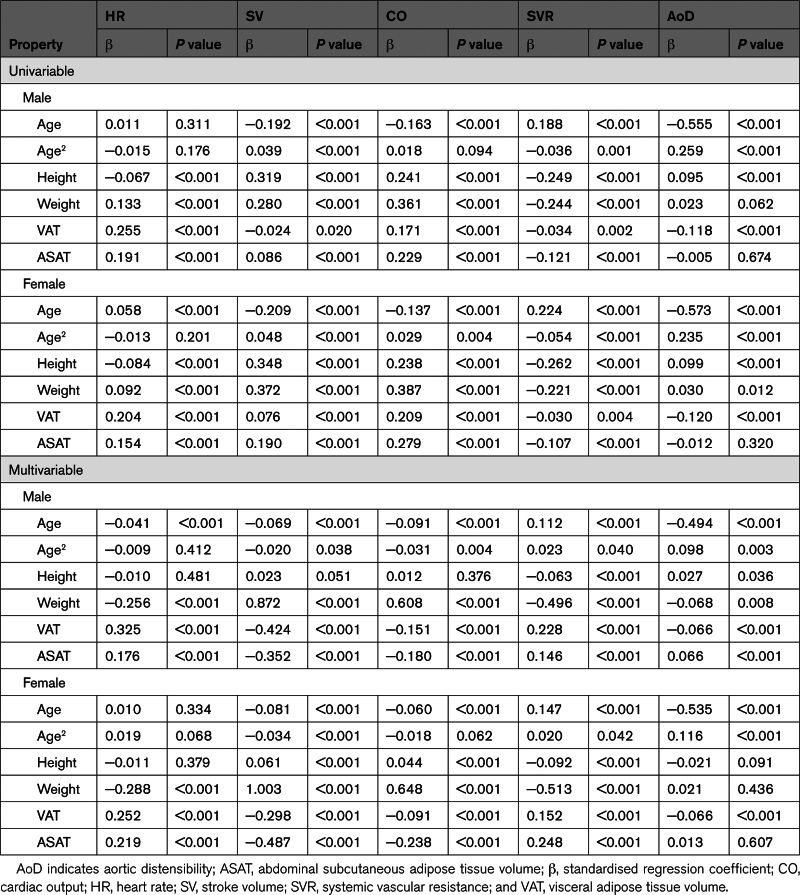
Univariable and Multivariable Regression Analysis of Associations Between Cardiovascular Properties and Measures of Body Composition

## DISCUSSION

The large UK Biobank imaging study provides an unparalleled resource to characterize the hemodynamics of raised BP and hypertension in the middle to older-aged UK population. The main finding is that an increase in CO is at least as important a cause of raised BP and hypertension as is an increase in SVR. This is at variance with the established view that primary hypertension results mainly from an increase in SVR.^7^ However, the evidence for this historical view comes mainly from studies that have compared groups of hypertensive compared with normotensive subjects, with some studies including hypertensive subjects with severe hypertension. In population studies in young adults, raised BP has been associated with an increase in CO or an increase in aortic stiffness (implying a decrease in AoD).^8–10^ In the present study, we find that all 3 factors, CO, SVR, and AoD contribute to raised BP in adults over the range 45 to 84 years. Furthermore, we detected an interaction with age such that CO was a proportionately more important determinant of elevated BP in younger people and SVR a more important determinant in older subjects. This is consistent with the observation of a raised CO being the main determinant of hypertension in children and young adults and both CO and SVR being implicated in hypertension in older adults in a recent meta-analysis comparing groups of hypertensive and normotensive subjects.^19^ Although CO and SVR determine MAP, PP is influenced by AoD and other factors. In the present study, we found that AoD was inversely associated with the level of BP, contributing to raised PP as well as raised MAP in the higher compared with lower BP groups. Of note the difference in AoD between higher and lower BP groups and between subjects with ISH and normotensive subjects was more marked in younger compared with older subjects and particularly so in women compared with men. This is surprising because aortic and large artery stiffening with reduced AoD is thought to be a particularly important cause of raised PP and ISH in the elderly.^20^ However, unlike MAP which is directly related to CO and SVR, there is a nonlinear inverse relationship between PP and AoD and there are many factors other than AoD that influence PP such as aortic flow velocity and the rate of rise of aortic flow and pressure.^5^ In the present analysis, we found a significant association of Umax with PP, consistent with a role of aortic flow in determining PP as expected from the physics of the circulation with the PP in early systole being determined to a first approximation by the Water Hammer equation^5^ and hence by the product of pulse wave velocity and Umax. However, unlike the contribution of CO and SVR to MAP, where the percentage change in MAP is equal to the sum of the percentage changes in CO and SVR, the exact contribution of AoD to PP is more difficult to quantitate. Nevertheless, the present results demonstrate the importance of aortic stiffening in increasing PP in younger subjects, a finding in keeping with the observation that aortic stiffening contributes to ISH in children and young adults.^9,10^

An important consideration when assessing hemodynamic properties such as CO and SVR is whether these should be normalized for body size and composition. As raised BP and hypertension are associated with greater adiposity, the role of adiposity is of particular importance. Adjustment of CO/SVR for adiposity is not appropriate when addressing the question as to whether the primary hemodynamic abnormality in hypertension is an increase in CO or SVR that may be in part secondary to adiposity but is important in determining whether an increase in CO or SVR could be mediated by adiposity. The detailed MRI measurements of VAT and ASAT available in this study allow us to examine the role of adiposity in more detail than has previously been possible. We find that after adjustment for body surface area, there was a significant negative association of CO with VAT and ASAT and SVR was positively associated with these measures of adiposity. However, the effect size was modest, and adjusting for body surface area, VAT, and ASAT made little difference to the contribution of CO and SVR (or AoD) to raised BP and hypertension.

The strength of the present study relates to the large sample size and accurate measures of CO provided by MRI. However, many limitations should be noted. Participants in UK Biobank may not be entirely representative of the wider UK population. Although BP measurement was carefully standardized, it was subject to the usual limitations of office BP measurement and, therefore, did not exclude white coat hypertension. Automated measurement of distensibility was achieved in only a subset of participants. Although the measurement is theoretically closely related to the gold-standard measure of aortic stiffness pulse wave velocity, and has been shown to have similar association to risk factors and similar predictive value for cardiovascular events as pulse wave velocity,^21^ its relationship to pulse wave velocity requires further validation. Strengths of associations of cardiovascular properties to BP components obtained by regression analysis should be interpreted with caution because regression coefficients may be influenced by BP components themselves being used in the calculation of the cardiovascular properties (eg, SVR=MAP/CO and AoD=strain/PP). Finally, conclusions about longitudinal trends cannot be made from a cross-sectional study of this nature.

### Perspectives

Previously reported findings of a raised CO being an important cause of hypertension in children and young adults,^8–10^ together with the observation in the present study that this persists into middle and older-age, have important implications for understanding the pathogenesis of hypertension and for its prevention and treatment. Rather than being a condition characterized by resistance vessel dysfunction, these findings suggest that environmental, renal, and neurohormonal factors that lead to intravascular volume expansion and an increase in sympathetic activity increasing SV and HR, respectively, are important in the pathogenesis of hypertension. Indeed, resistance vessel dysfunction could be secondary to an increase in BP as has been previously suggested and may be more prevalent in older subjects who have experienced raised BP of longer duration.^22^ It is notable that salt restriction and diuretics are 2 of the most effective lifestyle and pharmacological interventions for the prevention and treatment of hypertension. Although both may reduce SVR, they may also have a sustained effect to reduce CO. The relative action of diuretics on SVR and CO deserves further study using accurate measurements such as MRI and would inform the direction of future research in hypertension therapeutics.

In conclusion, raised BP and hypertension in the middle to older-aged UK population are associated with a raised CO. The pathogenesis of hypertension may be more strongly linked to factors that elevate CO than previously recognized; interventions that target these factors may be effective in preventing and treating hypertension.

## ARTICLE INFORMATION

### Sources of Funding

This work was supported by grants from the British Heart Foundation (PG/17/50/32903) and Medical Research Council (MR/M01656Q/1).

### Disclosures

None.

## Supplementary Material
