# Supplementary material for: Hemodynamic Determinants of Elevated Blood Pressure and Hypertension in the Middle to Older-Age UK Population: A UK Biobank Imaging Study
Source: Hypertension. 2023 Sep 7;80(11):2473–84. doi: 10.1161/HYPERTENSIONAHA.122.20969 (PMC10876164; doi:10.1161/HYPERTENSIONAHA.122.20969)
Supplement: Supplementary file 1 [file hyp-80-2473-s001.docx]

**Supplemental Material**

**Hemodynamic determinants of elevated blood pressure and hypertension in the middle to older-age UK population: a UK Biobank imaging study**

Ye Li^1^

Emily Chan^2^

Esther Puyol-Antón^2^

Bram Ruijsink^2^

Marina Cecelja^1^

Andrew P. King^2^

Reza Razavi^2^

Phil Chowienczyk^1^

^1^ King’s College London British Heart Foundation Centre, Department of Clinical Pharmacology, St. Thomas’ Hospital

^2^ School of Bioengineering and Imaging Science, King’s College London

**Correspondence to:**

Prof PJ Chowienczyk*,* Department of Clinical Pharmacology*,* St Thomas’ Hospital Lambeth Palace Road London SE1 7EH*,* UK Tel: + 44 207 1881504*,* Fax: + 44 207 4012242*,* E-mail: [phil.chowienczyk@kcl.ac.uk](mailto:phil.chowienczyk@kcl.ac.uk)

Table S1: Demographics, blood pressure and hemodynamic measures in normotensive subjects and subjects with isolated systolic hypertension and isolated diastolic hypertension

|  | BP groups | | | | | P value |
| --- | --- | --- | --- | --- | --- | --- |
|  | Normotensive | | ISH | | IDH |  |
| **Descriptive variables** | | | | | | |
| n= | 15,074 | 9,664 | | 479 | |  |
| Age (years) | 61±7 | 66±7 | | 59±7 | | <0.001 |
| Sex (male%) | 37.9 | 52.4 | | 57.4 | | <0.001 |
| Height (cm) | 169.6±9.38 | 170.2±9.43 | | 172.2±9.16 | | <0.001 |
| BMI (kg/m^2^) | 25.3±3.83 | 26.8±4.26 | | 28.5±5.31 | | <0.001 |
| Diabetic (%) | 2.18 | 6.15 | | 2.92 | | <0.001 |
| Current smoker (%) | 3.96 | 2.90 | | 4.19 | | <0.001 |
| Anti-hypertensive treatment (%) |  | 24.4 | | 21.5 | | <0.001 |
| **Blood pressures** |  |  | |  | |  |
| SBP (mmHg) | 124±10 | 152±11 | | 134±4 | | <0.001 |
| DBP (mmHg) | 73±8 | 80±6 | | 92±3 | | <0.001 |
| MAP (mmHg) | 90±7 | 104±6 | | 106±2 | | <0.001 |
| pPP (mmHg) | 51±9 | 72±12 | | 42±5 | | <0.001 |
| cPP (mmHg) | 58±17 | 71±19 | | 57±17 | | <0.001 |
| **Hemodynamics** |  |  | |  | |  |
| HR | 61±10 | 63±10 | | 68±13 | | <0.001 |
| SV (ml) | 81.2±18.5 | 85.9±19.8 | | 80.8±18.4 | | <0.001 |
| CO (L/min) | 4.91±1.15 | 5.32±1.29 | | 5.41±1.30 | | <0.001 |
| SVR (mmHg.min/L) | 19.3±4.66 | 20.7±5.34 | | 20.7±5.05 | | <0.001 |
| AoD (10^-3^ mmHg^-1^) | 2.37±1.22 | 1.34±0.81 | | 2.42±1.14 | | <0.001 |

AoD, distensibility of aorta; BMI, body mass index; HR, heart rate; CO, cardiac output; DBP, diastolic blood pressure; IDH, isolated diastolic hypertension; ISH, isolated systolic hypertension; MAP, mean arterial pressure; PP, pulse pressure; SBP, systolic blood pressure; SV, stroke volume; SVR, systemic vascular resistance.

Table S2: Characteristics of participants in UK Biobank imaging study including sub-groups with aortic distensibility and body composition measures

|  | Whole group | | AoD measurements | | Body composition measurements | P value |
| --- | --- | --- | --- | --- | --- | --- |
| **Descriptive variables** | | | | | | |
| n= | 31,112 | 21,178 | | 19,645 | |  |
| Age (years) | 63±8 | 62±7 | | 62±7 | | <0.001 |
| Sex (male%) | 46.3 | 49.3 | | 46.4 | | <0.001 |
| Height (cm) | 170.1±9.45 | 170.6±9.41 | | 170.0±9.44 | | <0.001 |
| BMI (kg/m^2^) | 26.3±4.31 | 26.3±4.12 | | 26.4±4.27 | | <0.001 |
| Diabetic (%) | 4.44 | 4.25 | | 4.18 | | <0.001 |
| Smoking (%) | 3.43 | 3.58 | | 3.66 | | <0.001 |
| **Blood pressures** | | | | | | |
| SBP (mmHg) | 138±18 | 137±18 | | 137±18 | |  |
| DBP (mmHg) | 79±10 | 78±10 | | 79±10 | |  |
| MAP (mmHg) | 98±12 | 98±12 | | 98±12 | | <0.001 |
| PP (mmHg) | 59±14 | 58±14 | | 59±14 | | <0.001 |
| **Hemodynamics** | | | | | | |
| HR (bpm) | 62±10 | 62±10 | | 63±10 | | <0.001 |
| SV (ml) | 83.4±19.2 | 84.4±19.0 | | 83.6±19.3 | | <0.001 |
| CO (L/min) | 5.15±1.27 | 5.16±1.24 | | 5.18±1.27 | | <0.001 |
| SVR  (mmHg.min/L) | 20.1±5.15 | 19.9±5.01 | | 19.9±5.07 | | <0.001 |

BMI, body mass index; HR, heart rate; CO, cardiac output; DBP, diastolic blood pressure; MAP, mean arterial pressure; PP, pulse pressure; SBP, systolic blood pressure; SV, stroke volume; SVR, systemic vascular resistance.

Table S3: Univariable and multivariable regression analysis between hemodynamic and body composition measures

|  | HR | | SV | | CO | | SVR | | AoD | |
| --- | --- | --- | --- | --- | --- | --- | --- | --- | --- | --- |
| Univariable | **β** | P value | **β** | P value | **β** | P value | **β** | P value | **β** | P value |
| **Male** |  | |  | |  | |  | |  |  |
| Age | 0.011 | 0.311 | -0.192 | <0.001 | -0.163 | <0.001 | 0.188 | <0.001 | -0.555 | <0.001 |
| BSA | 0.081 | <0.001 | 0.343 | <0.001 | 0.376 | <0.001 | -0.285 | <0.001 | 0.050 | <0.001 |
| VAT | 0.255 | <0.001 | -0.024 | 0.020 | 0.171 | <0.001 | -0.034 | 0.002 | -0.118 | <0.001 |
| ASAT | 0.191 | <0.001 | 0.086 | <0.001 | 0.229 | <0.001 | -0.121 | <0.001 | -0.005 | 0.674 |
| **Female** |  | |  | |  | |  | |  |  |
| Age | 0.058 | <0.001 | -0.209 | <0.001 | -0.137 | <0.001 | 0.224 | <0.001 | -0.573 | <0.001 |
| BSA | 0.046 | <0.001 | 0.432 | <0.001 | 0.406 | <0.001 | -0.279 | <0.001 | 0.059 | <0.001 |
| VAT | 0.204 | <0.001 | 0.076 | <0.001 | 0.209 | <0.001 | -0.030 | 0.004 | -0.120 | <0.001 |
| ASAT | 0.154 | <0.001 | 0.190 | <0.001 | 0.279 | <0.001 | -0.107 | <0.001 | -0.012 | 0.320 |
| Multivariable | **β** | P value | **β** | P value | **β** | P value | **β** | P value | **β** | P value |
| **Male** | | | | | | | | |  |  |
| Age | -0.031 | 0.006 | -0.082 | <0.001 | -0.094 | <0.001 | 0.117 | <0.001 | -0.549 | <0.001 |
| BSA | -0.175 | <0.001 | 0.606 | <0.001 | 0.418 | <0.001 | -0.406 | <0.001 | -0.009 | 0.527 |
| VAT | 0.283 | <0.001 | -0.292 | <0.001 | -0.061 | <0.001 | 0.175 | <0.001 | -0.088 | <0.001 |
| ASAT | 0.112 | <0.001 | -0.139 | <0.001 | -0.029 | 0.081 | 0.048 | 0.004 | 0.032 | 0.044 |
| **Female** | | | | | | | | |  |  |
| Age | 0.014 | 0.199 | -0.084 | <0.001 | -0.060 | <0.001 | 0.148 | <0.001 | -0.564 | <0.001 |
| BSA | -0.186 | <0.001 | 0.686 | <0.001 | 0.452 | <0.001 | -0.441 | <0.001 | -0.005 | 0.757 |
| VAT | 0.214 | <0.001 | -0.220 | <0.001 | -0.050 | 0.001 | 0.130 | <0.001 | -0.068 | <0.001 |
| ASAT | 0.129 | <0.001 | -0.168 | <0.001 | -0.030 | 0.084 | 0.134 | <0.001 | 0.032 | 0.069 |

AoD, aortic distensibility; ASAT, abdominal subcutaneous adipose tissue; BSA, body surface area; CO, cardiac output; HR, heart rate; SV, stroke volume, SVR, systemic vascular resistance; VAT, visceral adipose tissue.


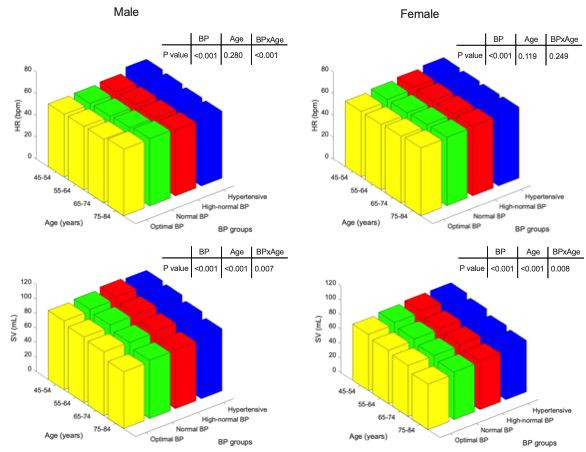


Figure S1: Cardiovascular variables HR and SV in UK Biobank stratified according to age and BP group. P values refer to significant differences in mean values of variables between age and BP groups and the interaction between age and BP (age x BP).


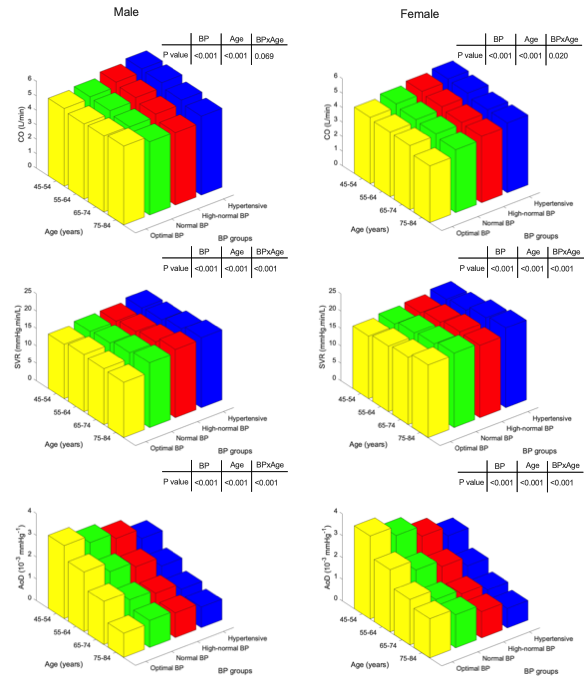


Figure S2: Cardiovascular variables CO, SVR and distensibility in UK Biobank stratified according to age and BP group. CO and distensibility adjusted by BSA + VAT + ASAT, SVR calculated as MAP/(CO adjusted). P values refer to significant differences in mean values of variables between age and BP groups and the interaction between age and BP (age x BP).


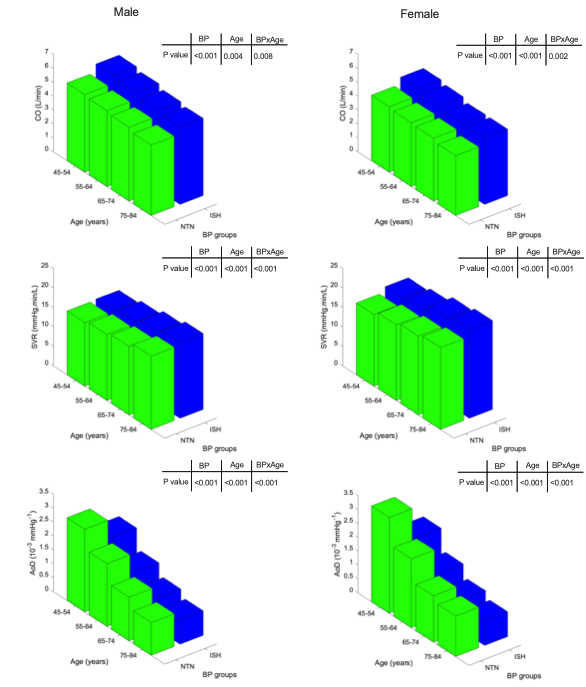


Figure S3: Cardiovascular variables CO, SVR and AoD in UK Biobank stratified according to age and BP group (BP grouped as normotensive and ISH). CO and distensibility adjusted by BSA + VAT + ASAT, SVR calculated as MAP/(CO adjusted). P values refer to significant differences in mean values of variables between age and BP groups and the interaction between age and BP (age x BP).
