# Supplementary material for: Hemodynamic Determinants of Elevated Blood Pressure and Hypertension in the Middle to Older-Age UK Population: A UK Biobank Imaging Study
Source: Hypertension. 2023 Sep 7;80(11):2473–84. doi: 10.1161/HYPERTENSIONAHA.122.20969 (PMC10876164; doi:10.1161/HYPERTENSIONAHA.122.20969)
Supplement: Supplementary file 2 [file hyp-80-2473-s002.pdf]

*** Equity Diversity and Inclusion Checklist**

Does this study focus on research involving differences in sex, gender, race/ethnicity, or other demographic categories or any other topic that should be reviewed through a lens of equity, diversity, and inclusion?

Yes

Disparities Research Reporting

Does this manuscript focus on reporting health differences by race and/or ethnicity? If so, a completed Disparities Research checklist [link] should be provided as a supplemental file.

No

Demographic Terminology

Does this manuscript adhere to the AMA's guidance for appropriate terminology for demographic information [PDF]? If historical terminology is used, is the usage explained?

NA

Sex, Gender, and Race/Ethnicity Specific Results

Are sex, gender, and race/ethnicity specific results of the study's primary outcomes reported regardless of whether there are significant differences by sex or race? If not, the reason for not reporting these results should be provided in the manuscript.

Yes

Demographic Representation for Clinical Research

Were steps taken to ensure the study is demographically representative, and if not, is the focus on one group or exclusion of other groups scientifically and conceptually justified?

Yes

Date completed: 08/01/2023 10:38:54
User pid: 18875
